# Molecular Characterization of the Complete Coding Sequence of Olive Leaf Yellowing-Associated Virus

**DOI:** 10.3390/plants9101272

**Published:** 2020-09-27

**Authors:** Ana Belén Ruiz-García, Thierry Candresse, Celia Canales, Félix Morán, Carlos Machado de Oliveira, Edson Bertolini, Antonio Olmos

**Affiliations:** 1 Instituto Valenciano de Investigaciones Agrarias (IVIA), 46113 Moncada, Spain; ana.belen.ruiz@uv.es (A.B.R.-G.); canales_cel@externos.gva.es (C.C.); moran_fel@gva.es (F.M.); 2 University Bordeaux, INRAE, UMR 1332 Biologie du Fruit et Pathologie, Equipe de Virologie, 33882 Villenave d’Ornon, France; thierry.candresse@inrae.fr; 3 Faculdade de Agronomia, Universidade Federal do Rio Grande do Sul (UFRGS), Porto Alegre 91540-000, Brazil; carlos_machado@icloud.com (C.M.d.O.); edson.bertolini@ufrgs.br (E.B.)

**Keywords:** *Closteroviridae*, thaumatin-like protein, genome, high-throughput sequencing

## Abstract

Genome organization and phylogenetic relationships of olive leaf yellowing-associated virus (OLYaV) with other members of the *Closteroviridae* family were determined. The complete coding sequence of OLYaV was obtained by high throughput sequencing of total RNA from a 35-year-old olive tree (cv. Zarzaleña) from Brazil, showing olive leaf yellowing disease and deformations in the wood. This represents the first report of OLYaV in this country. A genomic sequence of 16,700 nt containing 11 open reading frames (ORFs) was recovered, representing the complete virus coding capacity. The knowledge of the nucleotide sequence of the genome including the gene that codes the coat protein will facilitate the development of diagnostic tests, which are limited so far to PCR-based methods targeting the HSP70h gene. Interestingly, a thaumatin-like protein (ORF2), previously reported in other unassigned viruses in the *Closteroviridae* family, persimmon virus B and actidinia virus 1, was identified in the OLYaV genome. Phylogenetic analysis of shared proteins (ORF1a, ORF1b, HSP70h, HSP90h and CP) with all members of the *Closteroviridae* family provides new insight into the taxonomic position of these three closteroviruses and suggests they could represent a new genus in the family.

## 1. Introduction

Olive yellowing leaf-associated virus (OYLaV) was reported for the first time in Italy on cv. Biancolilla from Sicily, in plants showing bright chrome yellow discolorations of the leaves [1]. Partial characterization of its HSP70 gene identified the virus as a member of the family *Closteroviridae.* Its presence was confirmed in olive and in unidentified species of pseudococcid mealybugs and the psyllid *Euphyllura oliviana* fed on an infected cv. Biancolilla plant [1]. The partial genome information available for OLYaV (NC_043417) corresponds to only 4605 nucleotides containing partial ORF1b (RdRp), ORF2 (21kDa), ORF3 (7kDa), ORF4 (HSP70h) and the 5′-end of ORF 5 (HSP90h) [2], and a subsequent extension of 854 nucleotides in the HSP90h gene towards the 3′-end (AJ844555) [3]. The development and application of diagnostic methods by RT-PCR of specific regions of the HSP70 gene [1,4] has revealed that OLYaV is one of the most widespread olive viruses with different levels of incidence: California (USA) (93%) [5], Italy (64–21%) [6,7,8], Tunisia (49%) [9], Lebanon (24%) [10], Syria (15%) [11], Greece (5%) [12] and Albania (2%) [13]. In addition, studies of OLYaV HSP70 gene diversity revealed a high divergence in this region of samples collected in clonal germplasm repositories in California and Greece [5,12]. To date OLYaV remains as an unassigned species in the family *Closteroviridae* because more biological and molecular data is needed for its classification and because the available partial genome data do not allow one to clearly assign it to any of the genera in the family [14,15]. Initially, the closterovirus name was assigned to an unusual group of elongated plant viruses [16], reflecting their peculiar morphological characteristics as compared to other elongated plant viruses known at that moment. This led to the creation of the *Closterovirus* (closter in Greek for thread) genus, the first recognized species being beet yellows virus (BYV) and citrus tristeza virus (CTV). Carnation necrotic fleck virus (CNFV) was later included [17]. Further biological and molecular characterization efforts led to the description of more species, their structuring in several genera and to the creation of the *Closteroviridae* family. The molecular organization, evolution and taxonomy of closteroviruses have been discussed in several reviews [18,19,20,21]. Thus, the family *Closteroviridae* is now divided into four genera; *Closterovirus*, *Ampelovirus*, *Crinivirus* and the relatively recently created genus *Velarivirus*, in which forty-nine species are classified. Seven other species in the family remain unassigned to a genus: *Blueberry virus A* (BVA), *Actidinia virus 1* (AV1) and *Persimmon virus B*, for which complete genomes are available; *Mint vein banding-associated virus* (MVBaV) and OLYaV, for which only partial genomic sequences are available; and *Alligatorweed stunting virus* and *Megakepasma mosaic virus* without available nucleotide sequences [14]. Among these species, OLYaV is most closely related to PVB [22] and AV1 [23], as phylogenetic analyses based on the partial OLYaV genomic sequence cluster these three species together. In addition, PVB and AV1 share a common genomic organization with the ORF1ab, followed by HSP70, HSP90 and CP. They also lack a minor CP (CPm) and both species code for a thaumatin-like protein, unreported for other viruses of the family. In this study the near complete genome sequence of OYLaV, including its full coding potential, was obtained by high throughput sequencing (HTS) of total RNA from a 35-year-old olive tree (cv. Zarzaleña) from Brazil, a country where the virus had not been previously reported. The knowledge of the complete sequence will facilitate the development of diagnostic methods, expanding the possibilities to the detection of other genomic regions such as the CP gene. In addition, the phylogenetic analysis of ORF1a, ORF1b (RdRp), HSP70, HSP90 and CP with their homologues in other representative species of the *Closteroviridae* family opens new possibilities in the taxonomical classification of OLYaV.

## 2. Results

### 2.1. Genome Organization of Olive Yellowing Leaf-Associated Virus

#### 2.1.1. Bioinformatic Analysis

A nearly complete genomic sequence of OLYaV, isolate CS1 (deposited in GenBank under the MT809205 accession number) was recovered by HTS. A total of 51,587,867 reads were obtained after trimming of adapters and the quality control of the sequences. A host genome subtraction step was performed using the *Olea europaea* Oe6 scaffolds [24] and *Olea europaea subsp. europaea* complete plastid genome (NC_015401), resulting in 9714,650 reads remaining for the subsequent analysis. *De novo* assembly by CLC Genomics Workbench 10.1.1. (Qiagen, Hilden, Germany) allowed the recovery of a long contig of 16,700 nucleotides covered by 17,764 reads and with an average coverage of 128x. A BLASTN analysis with an e-value of 1.05 × 10^−151^ was obtained with the partial sequence of HSP70h of the isolate 7Oblonga3 from USA (HQ286495). The 98 nucleotides of the 5′ untranslated region (UTR) were covered with 45 reads of an average length of 121 nt and with the 10 extreme 5′ nucleotides covered by 11 non-redundant reads. The 159 nucleotides of the 3′UTR were covered by 172 reads of an average length of 121 nt, while the 10 extreme 3′ nucleotides were covered by 25 non-redundant reads, suggesting in both cases that the genomes ends have been reached or that minimal non-coding sequences were missing.

#### 2.1.2. Genomic Structure of OLYaV-CS1

**Figure 1:**
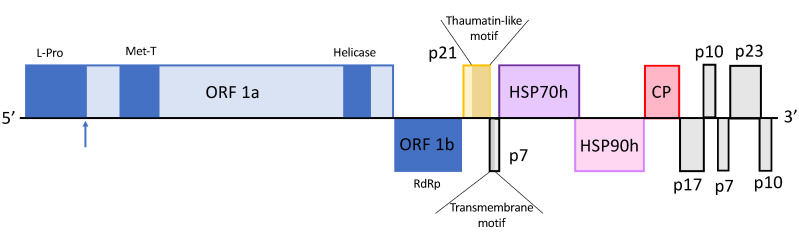
Genome organization of olive leaf yellowing-associated virus CS1 isolate. L-Pro: papain-like leader protease; Met-T viral methyltransferase domain; Helicase: viral helicase domain; RdRp: RNA dependent RNA polymerase. Blue arrow indicates tentative L-Pro cleavage site position.

The analysis of the genome structure of the isolate OLYaV-CS1 predicts 11 ORFs encoding proteins, some of which have homologies with those of other *Closteroviridae* members (ORF 1a and b, ORF2-thaumatin-like protein, ORF4-HSP70h, ORF5-HSP90h and ORF6-CP) while others have no homologous counterpart in the GenBank database, ORF3-p7, ORF7-p17, ORF8-p10, ORF9-p7, ORF10-p23 and ORF11-p10 (Figure 1).

- 5′UTR (1–98 nt)

**Figure 2:**
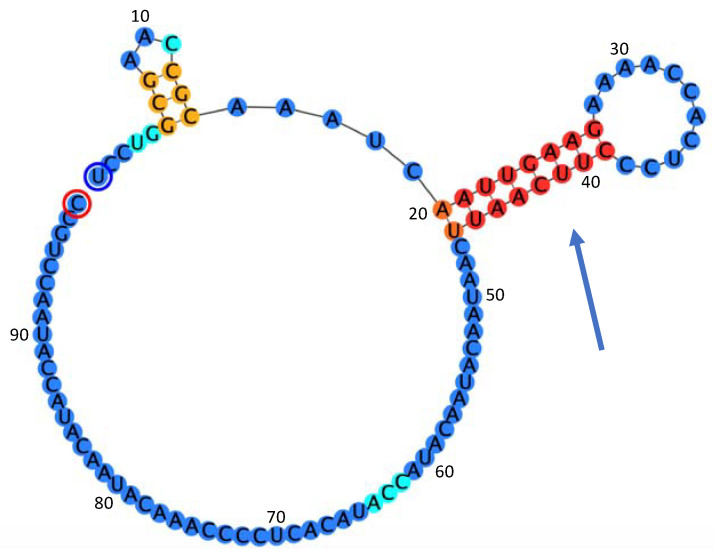
Prediction of the 5′UTR secondary structure computed by RNAfold implemented in Geneious Prime 2020. The blue arrow indicates the stem-loop that is predicted to remain stable up to 64 °C with a free energy of the thermodynamic ensemble ∆G = −2.58 kcal/mol in 1 M NaCl. The color of the nucleotides is related to the base pair probabilities provided by RNAFold: red, very high; orange, high; blue, low.

Computational analysis of the 5′UTR by RNAfold implemented in Geneious Prime 2020 (Biomatters Ltd., Auckland, New Zealand) based on Vienna package 2.4.15 (Figure 2) reveals the presence of two stem-loops, one at position 6–14 nt and another one at position 20–46 nt, using Turner [25] or Andronescu [26] as energy models. The predicted folding has a free energy of ∆G= −7.88 kcal/mol at 37 °C in 1 M NaCl. Both stem-loops were stable up to 58 °C using Turner or Andronescu models, with the largest stem-loop (20–26 nt) remaining stable up to 64 °C (∆G= −2.58 in 1 M NaCl) suggesting a possible involvement of this secondary structure in the virus replication.

- ORF1ab (nt 99–9792)

1. ORF1a (nt 99–8285)

**Figure 3:**
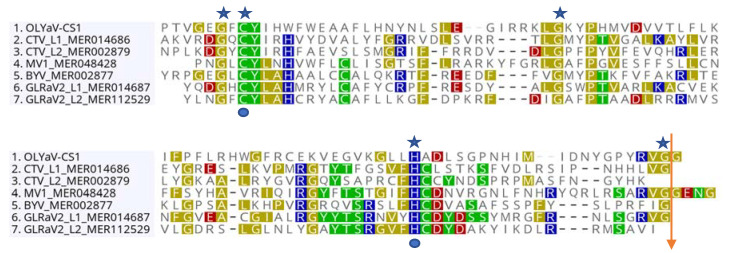
Alignment of the C-terminal region of papain-like leader proteases from different members of the *Closteroviridae* family. CTV: citrus tristeza virus; MV1: mint virus 1; BYV: beet yellows virus; GLRaV2: grapevine leafroll associated virus 2. Invariant residues are labelled with a blue star above the alignment. The predicted catalytic cysteine and histidine residues are labelled with a blue circle below the alignment. The orange arrow indicates the proposed cleavage site.

ORF1a codes for a multifunctional protein that contains a papain-like leader protease domain and conserved domains for a methyltransferase and a helicase. The viral methyltransferase domain (pfam01660) is located at amino acids (aa) 738–1038, while the viral helicase domain (pfam01443) is located at aa 2402–2609. In order to identify the possible cleavage site of the papain-like leader protease, a multiple alignment of the amino acid sequences of papain-like proteinase domains from various representative members of the family *Closteroviridae* available in the MEROPS database (https://www.ebi.ac.uk/) was used (Figure 3). This confirmed the presence of the expected invariant residues (G, C, P, H, G) [27] and suggested a cleavage site for the OLYaV leader protease between the conserved G–G dipeptide at position 460–461 of the protein, giving an estimated protease of molecular mass of 51 kDa.

2. ORF1b (8287–9793 nt)

ORF1b codes for the virus RNA dependent RNA polymerase (RdRp) and, similar to other members of the family *Closteroviridae* [18], is expressed through a + 1 ribosomal frameshift. The ORF1b encoded protein is 501 aa long and has an estimated mass of 58 kDa. BLASTP analysis showed the higher percentage similarity (83.1%) with the partial RdRp sequence previously reported of OLYaV (YP_009666027) and with the proteins of two unassigned *Closteroviridae* members, persimmon virus B (PVB; YP_009112883; 50.7% aa identity) and actinidia virus 1 (AV1; YP_009407919, 47.6% aa identity).

**Figure 4:**
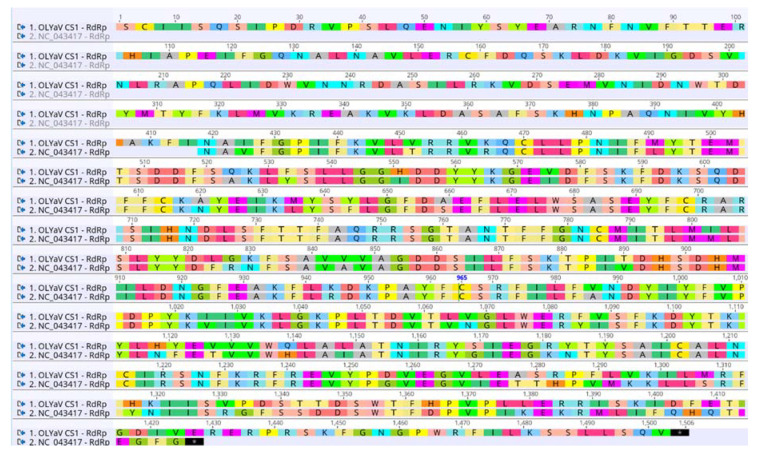
Amino acid alignment between the RdRp of OLYaV-CS1 and the RdRp of OLYaV NC_043417 isolates revealing a larger protein in the OLYaV-CS1 isolate. The numbers indicate the nucleotide position from the beginning of the ORF1b in the OLYaV CS1 isolate.

A large deletion was identified in the Brazilian isolate as compared to the partial sequence of OLYaV previously deposited in GenBank (NC_043417). Thus, when aligning OLYaV-CS1 with the reference isolate NC_043417, a deletion of 182 nt between positions 9689 and 9690 nt of the Brazilian isolate was found. The analysis by BLASTN of the 182 nt sequence present in the reference sequence NC_043417 did not identify any homologous sequence in GenBank. Sanger resequencing of a RT-PCR product covering this region in the Brazilian isolate confirmed that the nucleotide sequence recovered by HTS was correct. The indel is located in the reference sequence NC_043417 nine aa upstream of the ORF1b stop codon and extends to the initial part of the non-coding region between ORF1b and ORF2. As a consequence of this deletion, the RdRp of the two isolates have very different C-terminal ends and the protein of OLYaV-CS1 is 26 aa longer than that of the previously reported OLYaV isolate (Figure 4).

- ORF2 (nt 9861–10,430)

The analysis of the predicted 21 kDa protein (190 aa) encoded by ORF2, using the online tool for conserved protein domains search (CDD) at National Center for Biotechnology Information (NCBI) (https://www.ncbi.nlm.nih.gov/Structure/cdd/wrpsb.cgi?) revealed the presence of a thaumatin-like protein domain (superfamily smart00205) at aa positions 73 to 189. BLASTP analysis gave the highest percentages of identity with the previously reported OLYaV proteins (YP_009666028, 86.9% aa identity) and with proteins from AV1 (YP_009407925, 38.2% aa identity) and PVB (BAQ08238, 34.4% aa identity). These three viruses are the only members of the *Closteroviridae* family to possess such a protein with a thaumatin-like domain [22,23].

- ORF3 (nt 10,430–10,618)

ORF 3 codes for a 7 kDa protein of 62 aa. Analysis with InterPro Scan implemented in Geneious Prime 2020 suggest that this is a putative transmembrane protein, predicted by Phobious [28], with aa 1–5 predicted to be located externally, the region corresponding to aa 6–31 being the transmembrane region and aa 32–62 located in the cytoplasm. Similar small proteins with no clear amino acid homologies but predicted to be transmembrane proteins are found in many other *Closteroviridae* members.

- ORF4 (nt 10,615–12,390)

ORF4 codes for a heat shock protein 70 homolog (HSP70h) of 593 aa with a predicted molecular mass of 66 kDa. BLASTP analysis showed highest identities with the partial or complete proteins from other OLYaV isolates present in GenBank (81.6% to 94.2% aa identity). The most similar isolate was the 5Mission isolate from the USA and the most divergent was the OLYaV-Gr/Ms isolate from Greece. In all cases, the amino acid divergence level was lower than 25%, remaining within the species discrimination criterion and further confirming that the Brazilian CS1 isolate is indeed an OLYaV isolate. The closest *Closteroviridae* members are AV1 (37.6% aa identity) and PVB (35.2% aa identity).

- ORF5 (nt 12,290–13,837)

ORF5 codes for a heat shock protein 90 homologue (HSP90h) of 515 aa with a predicted molecular mass of 60 kDa. BLASTN analysis gave the highest homologies with the corresponding partial protein from OLYaV isolates, with identity values ranging from 82.6% to 79.1%; the most similar isolate being 0G9 from Italy and the most divergent one LM3, from Italy as well. In fact, only partial sequences from Italian isolates are available in GenBank. BLASTP analysis showed amino acid identity levels of 76.6–90.6% with these various OLYaV isolates. The closest *Closteroviridae* members with higher levels are again AV1 and PVB (respectively 24.5% and 24.1% aa identity).

- ORF6 (nt 13,904–14,605)

This ORF codes for a protein of 233 aa with an estimated molecular mass of 26 kDa. A conserved domain search found clear similarity to closteroviral coat proteins (superfamily pfam01785) in the region corresponding to aa 52–232. BLASTP analysis identified only similarity with the coat proteins of AV1 (range 27.3–29.9% aa identity for several isolates) and PVB (range 26.6–33.3% aa identity for several isolates).

- ORF7 (nt 14,606–15,085), ORF8 (nt 15,087–15,365), ORF9 (nt 15,375–15,569), ORF10 (nt 15,592–16,266) and ORF11 (nt 16,266–16,541).

These ORFs encode hypothetical proteins of respectively 160 aa (18 kDa), 93 aa (10 kDa), 65 aa (7 kDa), 225 aa (26 kDa) and 92 aa (10 kDa) of unknown function and without BLASTP-detectable identity with any protein in GenBank.

- 3′UTR (16,542–16,700 nt)

**Figure 5:**
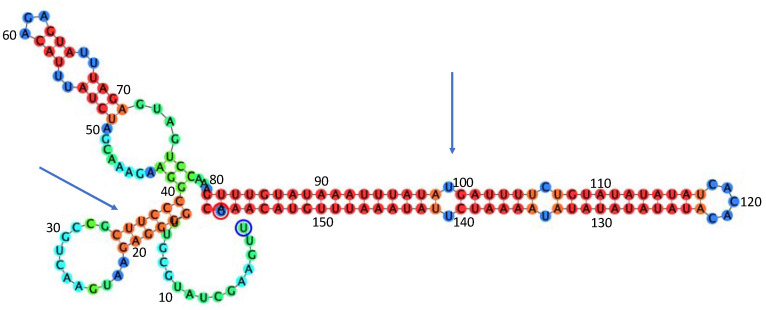
Secondary structure prediction for the 3′UTR computed by RNAfold implemented in Geneious Prime 2020. The blue arrow indicates the stem-loops that are predicted to remain stable up to 64 °C with a free energy of the thermodynamic ensemble ΔG = −18.38 kcal/mol in 1 M NaCl. The color of the nucleotides is related to the base pair probabilities provided by RNAFold: red, very high; orange, high; green, medium; blue, low.

Computational analysis of the 3′UTR by RNAfold implemented in Geneious Prime 2020 (Figure 5) revealed the presence of three predicted stem-loops, one at positions 15 to 40 (16,556 to 16,581 in the CS1 genome), a second one at positions 47 to 76 (16,588–16,617) and a larger one at positions 82 to 158 (16,623–16,669) with the last G free. Using Turner [25] or Andronescu [26] as energy models, a free energy of ∆G = −47.52 kcal/mol at 37 °C in 1 M NaCl was computed, with two stem-loops remaining stable at up to 64 °C (∆G = −18.38 kcal/mol in 1 M NaCl) using Turner or Andronescu energy models (Figure 5). Similar to the 5′UTR stem-loop, these two stem-loops are possibly involved in the virus replication.

### 2.2. Phylogenetic Analysis of Conserved Closteroviral Proteins (P1a, P1b, HSP70h, HSP90h, CP)

**Figure 6:**
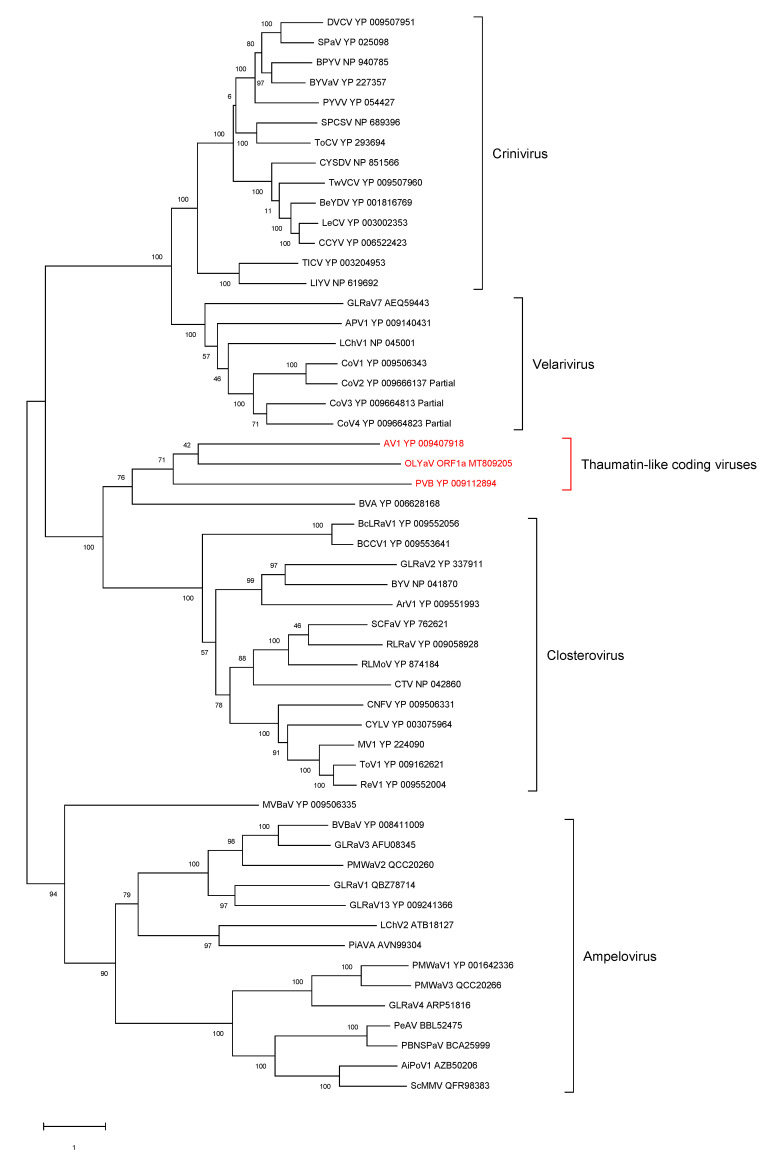
Maximum likelihood phylogenetic tree using the best substitution model by MEGA X of ORF1a from representative members of the family *Closteroviridae*. Proteins of the isolates are identified by their protein_id number. The scale bar shows the genetic distance. Bootstrap percentages (500 re-samples) are indicated on the branches.

**Figure 7:**
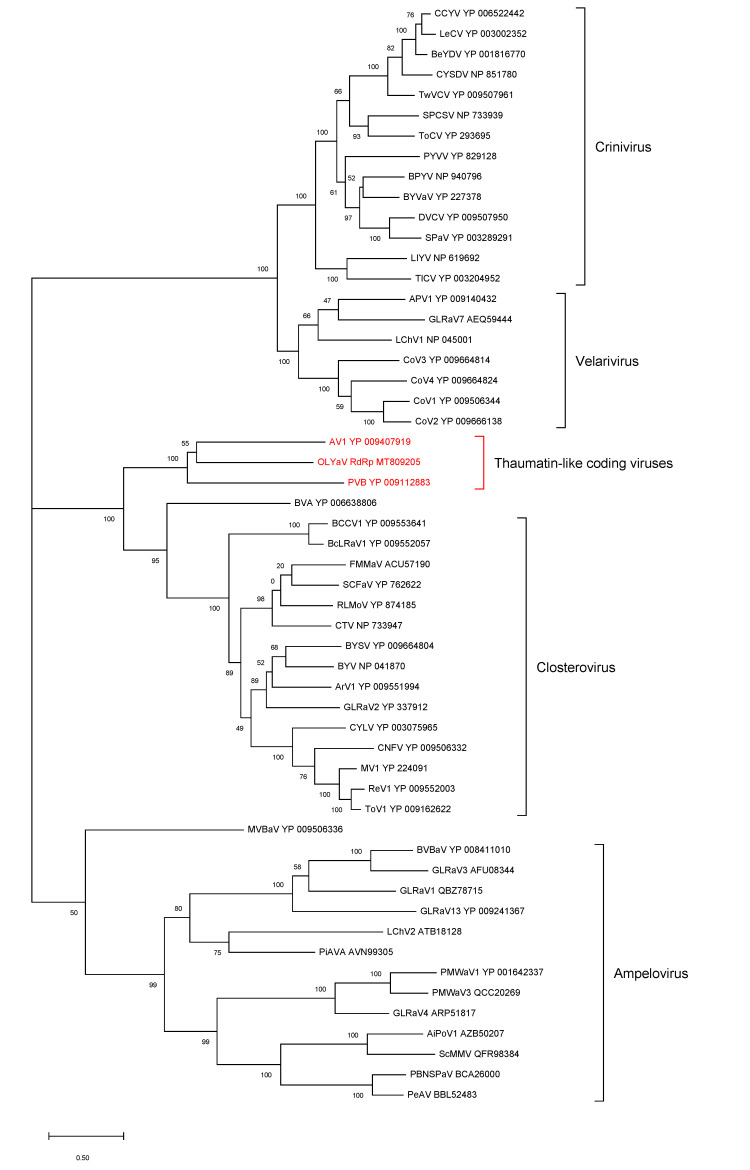
Maximum likelihood phylogenetic tree using the best substitution model by MEGA X of RdRp from representative members of the family *Closteroviridae*. Proteins of the isolates are identified by their protein_id number. The scale bar shows the genetic distance. Bootstrap percentages (500 re-samples) are indicated on the branches.

**Figure 8:**
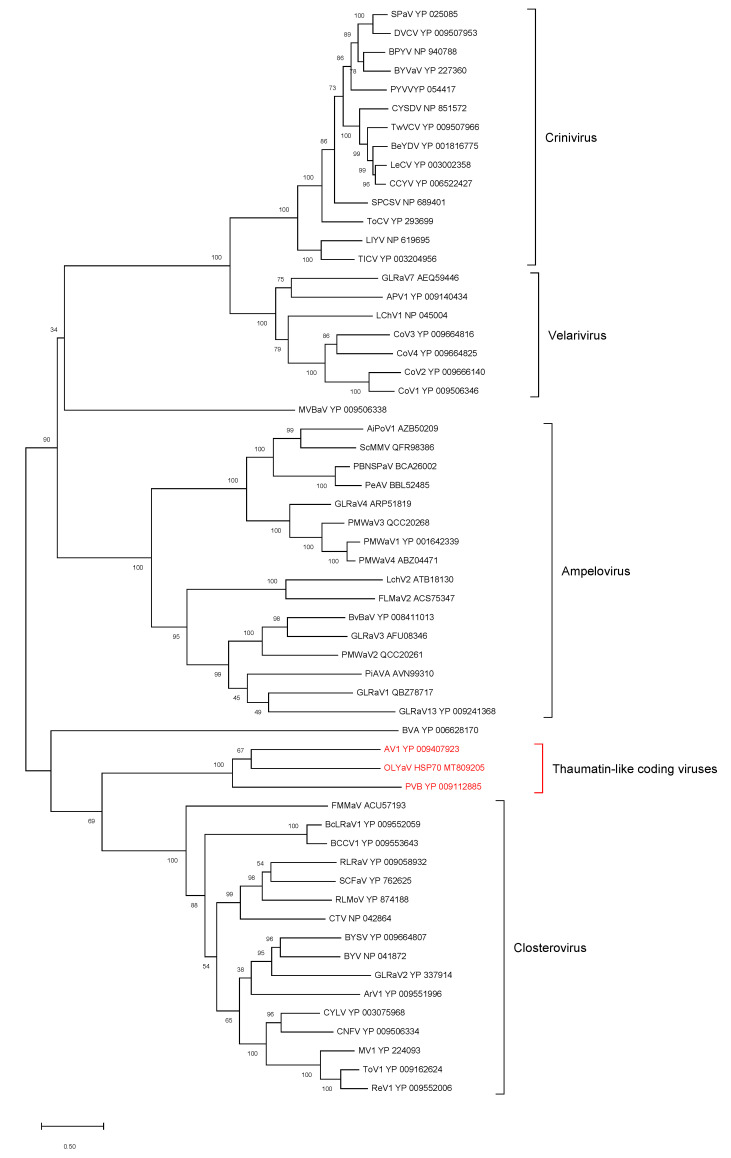
Maximum likelihood phylogenetic tree using the best substitution model by MEGA X of HSP70h from representative members of the family *Closteroviridae*. Proteins of the isolates are identified by their protein_id number. The scale bar shows the genetic distance. Bootstrap percentages (500 re-samples) are indicated on the branches.

**Figure 9:**
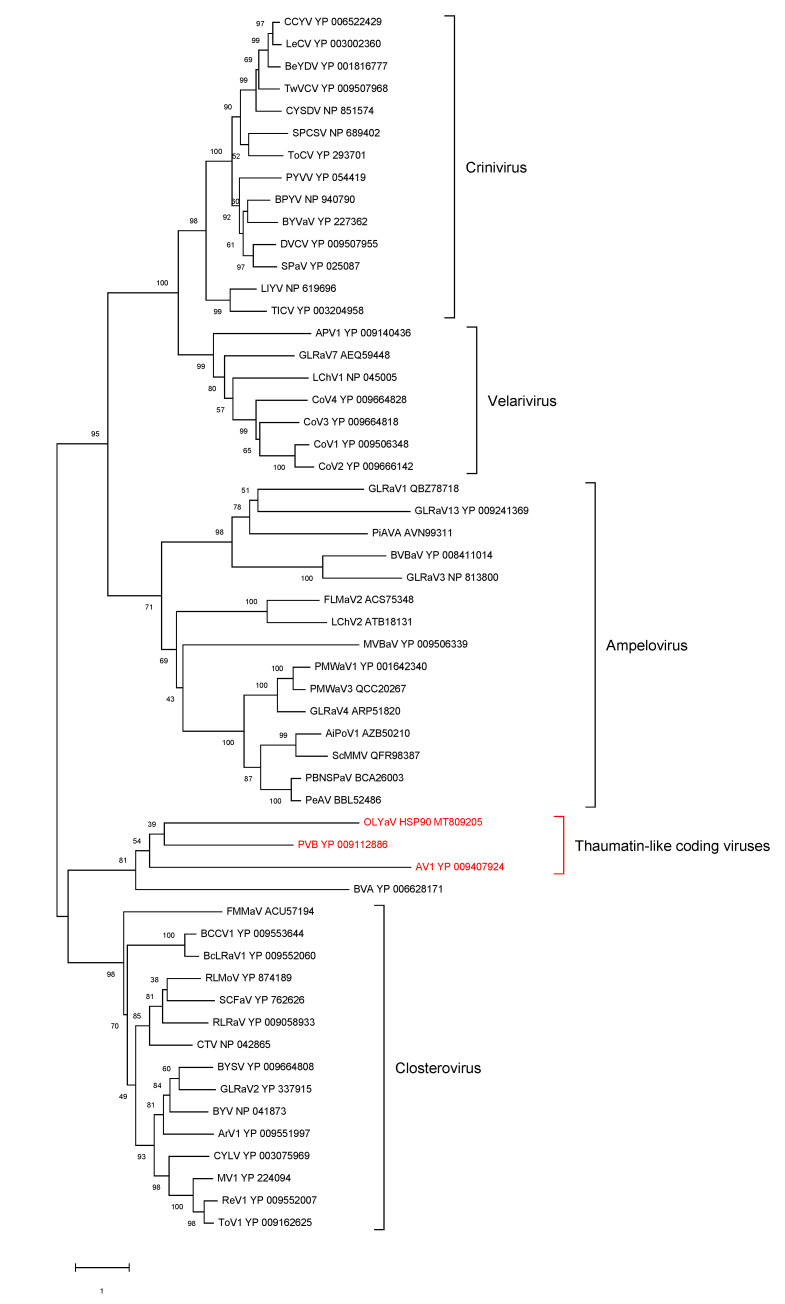
Maximum likelihood phylogenetic tree using the best substitution model by MEGA X of HSP90h from representative members of the family *Closteroviridae*. Proteins of the isolates are identified by their protein_id number. The scale bar shows the genetic distance. Bootstrap percentages (500 re-samples) are indicated on the branches.

**Figure 10:**
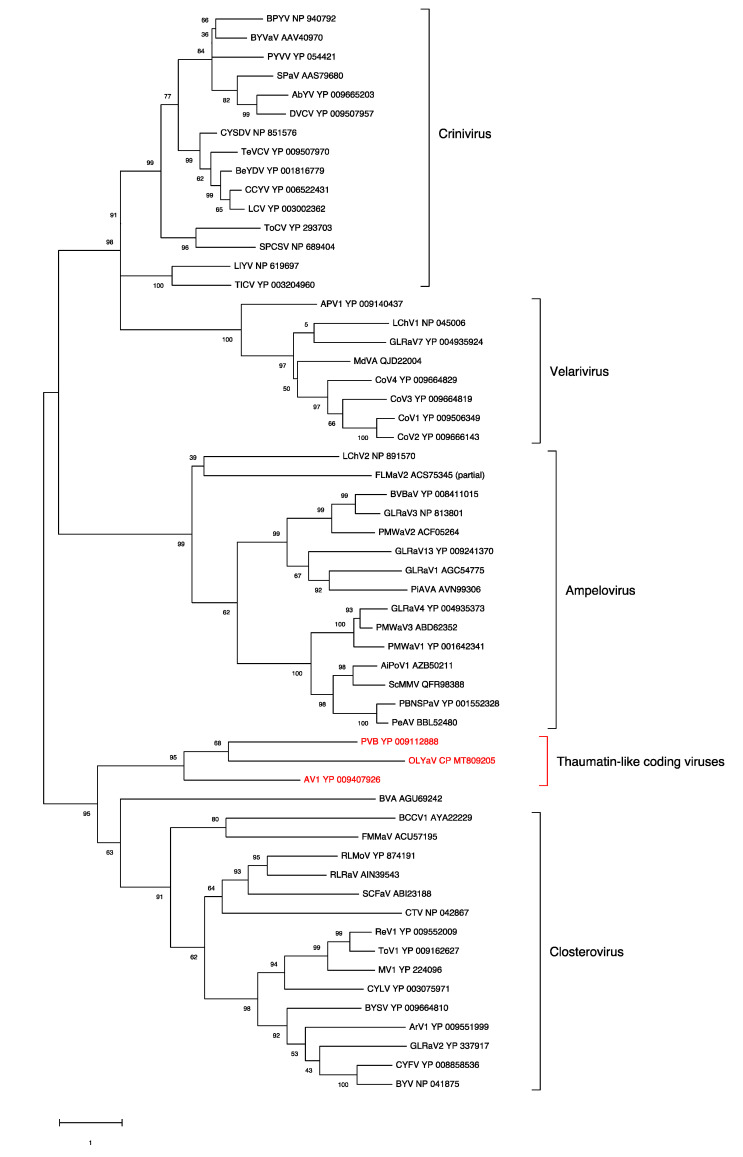
Maximum likelihood phylogenetic tree using the best substitution model by MEGA X of CP from representative members of the family *Closteroviridae*. Proteins of the isolates are identified by their protein_id number. The scale bar shows the genetic distance. Bootstrap percentages (500 re-samples) are indicated on the branches.

In all cases, the phylogenetic analyses were performed using the best substitution model computed by MEGA X [29] with the lowest BIC (Bayesian information criterion) and with 500 bootstraps (Figure 6, Figure 7, Figure 8, Figure 9 and Figure 10). For all conserved proteins, the best substitution model was the Le and Gascuel one [30], employing the same equilibrium frequencies (+F) with rates among sites gamma distributed and with invariant sites (G + I). Representative isolates from each *Closteroviridae* species were included. The results showed that OLYaV proteins clustered systematically and with high bootstrap support with those of AV1 and PVB, the two unassigned *Closteroviridae* members that are similar to OLYaV and that code for a thaumatin-like protein. This cluster of three viruses was systematically removed from the four known *Closteroviridae* genera, *Closterovirus*, *Ampelovirus*, *Velarivirus* and *Crinivirus*. The closest affinities were nevertheless with the *Closterovirus* genus, but with strong bootstrap support only in the P1b (RdRP) and CP trees. The unassigned blueberry virus A tended to cluster, albeit distantly, with the *Closteroviridae* genus, but grouped together with OLYaV, AV1 and PVB in the HSP90h tree, with solid (81%) bootstrap support.

The acronyms used in all maximum likelihood phylogenetic trees are as follows: AiPoV1: air potato virus 1; APV1: areca palm velarivirus 1; ArV1: arracacha virus 1; AV1: actinidia virus 1; BCCV1: blackcurrant-associated closterovirus 1; BcLRaV1: blackcurrant leafroll-associated virus 1; BeYDV: bean yellow disorder virus; BPYV: beet pseudo-yellows virus; BVA: blueberry virus A; BVBaV: blackberry vein banding associated virus; BYSV: beet yellow stunt virus; BYV: beet yellows virus; BYVaV: blackberry yellow vein-associated virus; BYVaV: blackberry yellow vein-associated virus; CCYV: cucurbit chlorotic yellows virus; CNFV: carnation necrotic fleck virus; CoV1: cordyline virus 1; CoV2: cordyline virus 2; CoV3: cordyline virus 3; CoV4: cordyline virus 4; CTV: citrus tristeza virus; CYLV: carrot yellow leaf virus; CYSDV: cucurbit yellow stunting disorder virus; DVCV: diodia vein chlorosis virus; FLMaV2: fig leaf mottle-associated virus 2; FMMaV: fig mild mottle-associated virus; GLRaV1: grapevine leafroll-associated virus 1; GLRaV2: grapevine leafroll-associated virus 2; GLRaV3: grapevine leafroll-associated virus 3; GLRaV4: grapevine leafroll-associated virus 4; GLRaV7: grapevine leafroll-associated virus 7; GLRaV13: grapevine leafroll-associated virus 13; LChV1: little cherry virus 1; LChV2: little cherry virus 2; LeCV: lettuce chlorosis virus; LIYV: lettuce infectious yellows virus; MV1: mint virus 1; MVBaV: mint vein banding-associated virus; OLYaV: olive leaf yellowing-associated virus; PBNSPaV: plum bark necrosis stem pitting-associated virus; PeAV: persimmon ampelovirus; PiAVA: pistachio ampelovirus A; PMWaV1: pineapple mealybug wilt-associated virus 1; PMWaV2: pineapple mealybug wilt-associated virus 2; PMWaV3: pineapple mealybug wilt-associated virus 3; PMWaV4: pineapple mealybug wilt-associated virus 4; PVB: persimmon virus B; PYVV: potato yellow vein virus; PYVV: potato yellow vein virus; ReV1: rehmannia virus 1; RLMoV: raspberry mottle virus; RLRaV: rose leaf rosette-associated virus; SCFaV: strawberry chlorotic fleck associated virus; SCFaV: strawberry chlorotic fleck associated virus; ScMMV: sugarcane mild mosaic virus isolate; SPaV: strawberry pallidosis-associated virus; SPCSV: sweet potato chlorotic stunt virus; TICV: tomato infectious chlorosis virus; ToCV: tomato chlorosis virus; ToV1: tobacco virus 1; TwVCV: tetterwort vein chlorosis virus

- ORF1a

- ORF1b (RdRp)

- HSP70h

- HSP90h

- CP

## 3. Discussion

*Closteroviridae* members have among the largest genomes of plant viruses (13,000 to nearly 19,000 nt) composed of one, two or three positive-sense, single-stranded RNA segments [14]. Currently there are four recognized genera in the family, *Closterovirus*, *Ampelovirus*, *Velarivirus* and *Crinivirus*. All the members of the family share some common features in their genome organization. They all have the ORF1ab, that contains one or two papain-like leader proteases, the conserved domains for methyltransferase and helicase and an RNA-dependent RNA polymerase expressed through +1 ribosomal frameshift. In addition, another characteristic of the family is the presence of a homolog to the cellular HSP70h and, in most members, the presence of a diverged, duplicate copy of the capsid protein called minor capsid protein (CPm). The genus demarcation criteria in the family include phylogenetic relationships in the RdRp, CP, HSP70h aa sequences, the identity of vectors, and the number of genomic RNAs, number and organization of ORFs and virion length [14]. In the currently recognized taxonomy, seven *Closteroviridae* species remain unassigned to a genus, including OLYaV, for which only partial sequences were available until now.

The OLYaV isolate CS1 herein reported was detected from a 35-year-old olive tree with typical leaf yellowing disease symptoms, as well as unusual woody cylinder deformations. Neither de novo assembling nor the mapping against other known olive viruses (cherry leaf roll virus, strawberry latent ringspot virus, cucumber mosaic virus, olive latent virus 1, olive latent virus 2, olive latent virus 3, olive latent ringspot virus, tobacco mosaic virus, olive vein yellowing-associated virus, tobacco necrosis virus or olive mild mosaic virus) were able to detect any of them. Thus, OLYaV is a candidate for the unusual woody cylinder symptoms as well as for the more typical leaf yellowing symptoms observed, although further studies are required to fully clarify this issue. The recovered sequence of OLYaV-CS1 included non-coding regions of respectively 98 nt (5′UTR) and 159 nt (3′UTR) that could not be further extended because no overlapping reads were found. Other closteroviruses such as BCCV1 (NC_040834) or BcLRaV1 (MH460557) have similar 5′UTR (97 nt) sizes and others such as BVA (NC_018519) or BYV (AF AF056575) present similar in 3′UTR (176 and 166 respectively) sizes. However, the genomes of the closest *Closteroviridae* members, AV1 and PVB, have somewhat longer non-coding regions: respectively 305 and 184 nt (5′UTR) and 404 and 282 nt (3′UTR). This suggests that the extreme genome ends of OLYaV may not have been reached. Nevertheless, the full coding potential of the OLYaV genome has been established.

The complete coding sequence of the genome herein reported for OLYaV-CS1, shows a genome structure typical of the *Closteroviridae* family. It contains 11 ORFs, among them the complex ORF1ab encoding for the three typical conserved domains, papain-like leader protease, methyltransferase and helicase, and the RdRp. ORF1a codes for only one papain-like leader protease, as other members of the family do, such as BYV, MV1, LChV1 or LIYV [21,27]. There was a large deletion in the ORF1b of the Brazilian isolate as compared to the reference sequence. Interestingly, this indel results in the coding of a larger RdRp for the CS1 isolate, because of the absence of the stop codon present in NC_043417. Thus, this deletion does not shorten the size of the RdRp in the Brazilian isolate that is indeed 26 aa larger than the predicted RdRp of the partial reference isolate NC_043417, highlighting the divergence of these two OLYaV isolates. Another interesting point is related to the nucleotide sequence of the HSP70h gene. This genomic region, currently used for OLYaV diagnosis [1,4], has been found to exhibit a high level of variability [5,12], which might represent a challenge when trying to design primers of high inclusiveness for OLYaV detection. In fact, the CS1 Brazilian isolate was not detected using previously reported detection methods, due to its divergence in this genomic region. The availability of the complete coding sequence of the virus will provide new possibilities to improve the inclusive detection of this pathogen.

Besides its significant phylogenetic remoteness from members of the four *Closteroviridae* genera, which had contributed to its unassigned species status, OLYaV exhibits two original genomic features. First, ORF2 encodes for a thaumatin-like protein and second it does not code for a CPm protein. Interestingly, these two genomic features are shared by two other unassigned *Closteroviridae* species, AV1 and PVB [22,23]. It is noteworthy that the thaumatin-like protein conserved between the three viruses is not placed in the same genomic position in the various viruses. In OLYaV, the thaumatin-like protein is coded by ORF2, located between ORF1b and ORF3 (putative transmembrane protein), whereas in PVB and AV1 it is coded by an ORF located between the HSP90 and CP genes. This viral thaumatin-like protein has been previously suggested to have a symbiotic interaction with the plant [23], as it is known that plant thaumatin-like proteins have antifungal activities [31], are involved in plant resistance to pathogens [32], and provide protection against abiotic stress [33]. The lack of CPm is another interesting feature, as it is known that this protein is common to *Closteroviridae* members and encapsidates the 600–700 5′-terminal nucleotides, and are believed to contribute to the initiation of encapsidation. There are however some exceptions, as the ampeloviruses with the smallest genomes, such as GLRaV-4 or PMWaV-1, do not appear to possess a CPm [14]. How these viruses initiate their encapsidation without CPm remains to be studied. It is also interesting to mention that there is no known vector to date for OLYaV, AV1 or PVB. It has been proposed that *Closteroviridae* evolved from a common ancestor and diverged with the adaptation to specific vectors; aphids in the case of closteroviruses, mealybugs in the case of ampeloviruses, whiteflies in the case of criniviruses [21].

The genome properties shared between PVB and AV1, the presence of a thaumatin-like protein, and the lack of a CPm, as well as the close phylogenetic relationship between them in the HSP70 genomic region, have been previously reported [22,23]. However, the lack of knowledge on the OLYaV genome had limited advances in the taxonomical classification of these three unassigned species. In this study, the completion of the full or near full genomic sequence of OLYaV has allowed us to discover the presence in OLYaV of two genomic features shared by these three species, a coding sequence for a thaumatin-like protein and the lack of a CPm, and to confirm a close phylogenetic relationship between OLYaV, AV1 and PVB in all *Closteroviridae* conserved proteins as these three viral species systematically cluster together with high bootstrap support. Taken together, the remoteness of these three viruses from extant *Closteroviridae* genera, their shared original genomic features and their phylogenetic relatedness provide clear markers and strong support for the creation of a novel genus within the family to gather these three species.

## 4. Materials and Methods

### 4.1. Plant Material

**Figure 11:**
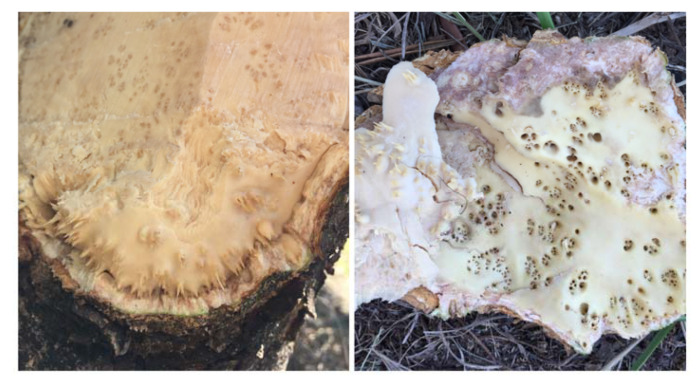
Wood deformations observed in the CS1 35-year-old olive tree cv. Zarzaleña.

Stems of a 35-year-old olive tree (CS1) cv. Zarzaleña, showing typical leaf yellowing disease symptoms and also unusual woody cylinder deformations like stem pitting and woody gall symptoms (Figure 11) were collected during the late spring season (11 November) in 2019 in Cachoeira do Sul, RS, Brazil (30°0′35″ S/52°52′06″ W).

### 4.2. Sample Preparation and HTS Analysis

Total RNA was purified from phloem scrappings using the Plant/Fungi total RNA purification kit (Norgen Biotek Corporation, Thorold, ON, Canada) according to the manufacturer’s protocol. Complementary DNA was synthesized and the sequencing library prepared using the TruSeq Stranded Total RNA LT Sample Prep Kit (Plant) and the library protocol TruSeq Stranded Total RNA Sample Prep Guide, Part #15031048 Rev. E. RNA quality control, library construction and sequencing in a NextSeq 500 platform (paired 2 × 150 nt) were performed at Macrogen Inc. (Seoul, Korea).

### 4.3. Bioinformatic Analysis of HTS Data

Reads were subjected to trimming of adapters and quality control using CLC Genomics Workbench v.10.1.1 (Qiagen Bioinformatics, Hilden, Germany). *Olea europaea* genome Oe6 scaffolds [19] and *Olea europaea* subsp. europaea plastid complete genome (NC_015401) were used for genome subtraction. The parameters used included match score 1, mismatch cost 2, insertion cost 3, deletion cost 3, length fraction 0.7 and similarity 0.8. De novo assembly was performed using CLC Genomics Workbench v.10.1.1 with automatic word size, automatic bubble size, minimum contig length of 200 nt, map reads back to contigs, mismatch cost 2, insertion cost 3, deletion cost 3, length fraction 0.7, similarity fraction 0.8 and updating contigs. De novo contigs were annotated by BLAST analysis (BLASTN/X) against local and online virus, viroids and nt/nr databases.

### 4.4. Analysis of the Strucure of the Genome of OLYaV

Geneious Prime 2020 was used to analyze the complete genome of the CS1 isolate. ORFs were determined with ORF finder included as a plugin in the software. The RNAfold plugin was used to predict the secondary structure of the 5′UTR and 3′UTR. Computational analysis with InterPro Scan implemented in the Geneious Prime 2020 and the online tool from the National Center for Biotechnology Information (NCBI) (https://www.ncbi.nlm.nih.gov/Structure/cdd/wrpsb.cgi?) were used to search predicted proteins for conserved domains. Nucleotide and amino acid percentages of identity were obtained using the online BLASTN and BLASTP tools from the National Center for Biotechnology Information (https://blast.ncbi.nlm.nih.gov/Blast.cgi).

### 4.5. Phylogenetic Analysis

For the phylogenetic analysis of the sequence determined in this study, a representative isolate of each species belonging to the family *Closteroviridae* available from the EMBL-EBI and GenBank databases was included. Alignment of conserved proteins, ORF1a, ORF1b, HSP70, HSP90 and CP were performed using the ClustaW implemented in MEGA X [29] using the pairwise and multiple alignments gap opening penalty 10.00 and gap extension penalty 0.10. The phylogenetic trees were constructed with the maximum likelihood algorithm implemented in MEGA X applying Le and Gascuel [30] aa substitution models and employing the same equilibrium frequencies (+F) with rates among sites gamma distributed with invariant sites (G + I) and 500 bootstrap replicates.

## 5. Conclusions

The genomic characterization of OLYaV, an unassigned member of the family *Closteroviridae,* revealed that it is closest to AV1 and PVB. These three viruses have so far remained unassigned in the family and share several common genomic features. They uniquely code for a thaumatin-like protein and do not encode a CPm duplicate. Phylogenetic analyses of the five broadly conserved closteroviral proteins showed that OLYaV, AV1 and PVB systematically cluster together, supporting their close phylogenetic relationship, suggesting that these unassigned species could be grouped together in a new genus inside the *Closteroviridae* family.
